# γδ T Cells Acquire Effector Fates in the Thymus and Differentiate into Cytokine-Producing Effectors in a Listeria Model of Infection Independently of CD28 Costimulation

**DOI:** 10.1371/journal.pone.0063178

**Published:** 2013-05-09

**Authors:** Renee M. Laird, Benjamin J. Wolf, Michael F. Princiotta, Sandra M. Hayes

**Affiliations:** Department of Microbiology and Immunology, State University of New York, Upstate Medical University, Syracuse, New York, United States of America; University of Oslo, Norway

## Abstract

Both antigen recognition and CD28 costimulation are required for the activation of naïve αβ T cells and their subsequent differentiation into cytokine-producing or cytotoxic effectors. Notably, this two-signal paradigm holds true for all αβ T cell subsets, regardless of whether they acquire their effector function in the periphery or the thymus. Because of contradictory results, however, it remains unresolved as to whether CD28 costimulation is necessary for γδ T cell activation and differentiation. Given that γδ T cells have been recently shown to acquire their effector fates in the thymus, it is conceivable that the contradictory results may be explained, in part, by a differential requirement for CD28 costimulation in the development or differentiation of each γδ T cell effector subset. To test this, we examined the role of CD28 in γδ T cell effector fate determination and function. We report that, although IFNγ-producing γδ T (γδ-IFNγ) cells express higher levels of CD28 than IL-17-producing γδ T (γδ-17) cells, CD28-deficiency had no effect on the thymic development of either subset. Also, following Listeria infection, we found that the expansion and differentiation of γδ-17 and γδ-IFNγ effectors were comparable between CD28^+/+^ and CD28^−/−^ mice. To understand why CD28 costimulation is dispensable for γδ T cell activation and differentiation, we assessed glucose uptake and utilization by γδ T cells, as CD28 costimulation is known to promote glycolysis in αβ T cells. Importantly, we found that γδ T cells express higher surface levels of glucose transporters than αβ T cells and, when activated, exhibit effector functions over a broader range of glucose concentrations than activated αβ T cells. Together, these data not only demonstrate an enhanced glucose metabolism in γδ T cells but also provide an explanation for why γδ T cells are less dependent on CD28 costimulation than αβ T cells.

## Introduction

The current paradigm for the activation of naïve αβ T cells and their subsequent differentiation into cytokine-producing or cytotoxic effectors is that two signals are required: one through the T cell antigen receptor (TCR) and the other through the co-stimulatory molecule, CD28. These two signals act together not only to prevent anergy [1]–[3], but also to promote cell survival [4], to activate the switch to glycolysis [5], [6], to stabilize cytokine gene transcripts [7], [8], and to regulate alternative splicing [9].

While most αβ T cells differentiate into effectors in the periphery, some αβ T cells subsets, such as Natural Killer T (NKT) cells and regulatory T (T_reg_) cells, acquire their effector functions in the thymus [10]–[14]. Despite the change in their site of differentiation, NKT and T_reg_ cells require CD28 costimulatory signals during their development in the thymus. Specifically, NKT cells require CD28 costimulation, following their selection, to expand and mature [15], [16], whereas T_reg_ cells require CD28 costimulation to activate the T_reg_ genetic program, which includes the expression of genes encoding Foxp3, GITR and CTLA-4 [17].

Due to conflicting results, it is unclear whether CD28 costimulation is also required for the activation and differentiation of γδ T cells. However, as the vast majority of these studies were conducted at a time when it was not known that γδ T cells have distinct effector fates and that acquisition of these fates occurs in the thymus [18], [19], it is possible that the conflicting results may be explained, in part, by each γδ T cell effector subset having a different requirement for CD28 costimulation, either during their development in the thymus or during their differentiation into effectors in the periphery. For this reason, we decided to re-evaluate the role of CD28 costimulation in the generation of γδ T cell effectors.

Here, we report that CD28 is differentially expressed between IFNγ-producing γδ T (γδ-IFNγ) cells and IL-17-producing γδ T (γδ-17) cells, with γδ-IFNγ expressing 2 to 3–fold more CD28 than γδ-17 cells. Despite this difference in expression, CD28 costimulation was not required to generate thymic and peripheral γδ-IFNγ and γδ-17 cells. Surprisingly, CD28 signaling was required to generate wild-type numbers of γδ thymocytes and γδ T cells. The reduction in the number of γδ lineage cells in CD28^−/−^ mice was not due to defects in either γδ lineage commitment or γδ thymocyte proliferation and survival, but instead was due to impaired proliferation of thymic progenitors. We also assessed the ability of CD28-deficient γδ T cells to differentiate into cytokine-producing effectors during infection with *Listeria monocytogenes* (Lm), and observed no difference in γδ T cell expansion and differentiation between infected CD28^+/+^ and CD28^−/−^ mice. Thus, these data not only indicate that CD28 is dispensable for γδ effector T cell development and differentiation but also highlight significant differences in the molecular requirements for the generation of αβ and γδ T cell effectors.

## Materials and Methods

### Ethics Statement

All research involving animals has been conducted according to the relevant national and international guidelines with respect to husbandry, experimentation and welfare. Mouse protocols were approved by the State University of New York (SUNY) Upstate Medical University Committee on the Humane Use of Animals (CHUA protocol numbers 262 and 281).

### Mice

C57BL/6J (CD28^+/+^), B6.129S2-Cd28^tm1Mak^/J (CD28^−/−^), and B6.129S2-Tcra^tmiMom^/J (TCRα^−/−^) mice were purchased from The Jackson Laboratory (Bar Harbor, ME, USA). B6-IL-23R-GFP knockin mice (IL-23R^gfp/+^) [20] were provided by M. Oukka (Seattle Children’s Research Institute, Seattle, WA, USA). C57BL/6-Vγ6/Vδ1 γδTCR transgenic (Tg) mice (γδTCR Tg; line 134) [21)] were provided by P.E. Love (NICHD, Bethesda, MD, USA). All mice used in this study were bred and maintained in the Department of Laboratory Animal Resources at SUNY Upstate Medical University under barrier conditions in accordance with the specifications of the Association for Assessment and Accreditation of Laboratory Animal Care. All mice were sacrificed at 5–8 weeks of age.

### Antibodies and Reagents

Monoclonal antibodies (mAbs) used for flow cytometric analysis and magnetic bead separation included anti-CD3 (145-2C11), anti-CD4 (RM4-5), anti-CD5 (53-7.3), anti-CD8α (53-6.7), anti-CD11b (M1/70), anti-CD19 (6D5), anti-CD25 (PC61), anti-CD27 (LG.3A10), anti-CD28 (E18), anti-CD44 (IM7), anti-CD49b (DX5), anti-CD117 (c-Kit; 2B8), anti-CCR6 (29-2L17), anti-IA^b^ (AF6-120.1), anti-NK1.1 (PK136), anti-Ly-6G (1A8), anti-TCRγδ (UC7-13D5), anti-Vγ1 (2.11), anti-Vγ4 (UC3-10A6), and anti-TCRβ (H57-597), which were purchased from BD Biosciences (San Jose, CA, USA), BioLegend (San Diego, CA, USA) or eBioscience (San Diego, CA, USA). Antibodies (Abs) used to measure surface expression of glucose transporter isoforms were anti-GLUT1 (N-20) and anti-GLUT3 (I-14), both purchased from Santa Cruz Biotechnology (Santa Cruz, CA, USA). mAbs used for intracellular staining were anti-Ki-67 (B56; BD Biosciences), anti-IL-17A (TC11-18H10.1; BioLegend), and anti-IFNγ (XMG1.2; BD Biosciences). Annexin V reagent was purchased from BioLegend.

### Flow Cytometric Analysis

Flow cytometric analysis for surface antigen expression was performed by pre-incubating cells with the anti-CD16/CD32 mAb for at least 10 minutes to block non-specific binding of immunoglobulins to Fc receptors, followed by staining with fluorochrome-conjugated mAbs against various surface antigens. For CD28 surface staining, PE-conjugated anti-CD28 mAb was used, and samples were run on a Becton Dickinson LSR II fitted with a 568 nm laser. For GLUT1 and GLUT3 surface staining, nonspecific binding was blocked by incubating cells with a mixture of donkey serum and the anti-CD16/CD32 mAb for at least 10 minutes. Cells were then stained with either the anti-GLUT1 or anti-GLUT3 Ab for 20 minutes, followed by staining with a fluorochrome-conjugated donkey anti-goat secondary antibody (Invitrogen, Carlsbad, CA, USA) for another 20 minutes.

Ki-67 and Annexin V staining was performed according to the manufacturer’s instructions (BD Biosciences). Intracellular staining for IL-17A and IFNγ was performed by first fixing cells in a final concentration of 1.5% formaldehyde for 10 minutes at 37°C. Fixed cells were then stained for surface antigens, permeabilized with Perm/Wash Buffer (BD Biosciences) for 20 minutes at 4°C, and then stained with mAbs against IL-17A and IFNγ.

For all experiments, 0.5 to 2.5 × 10^6^ cells were acquired on a BD LSR II or a BD LSRFortessa using FACSDiva software (BD Immunocytometry Systems, San Jose, CA USA). Data analysis was performed using FlowJo software (Tree Star, Inc., San Carlos, CA, USA). Dead cells were excluded from analysis based on forward and side scatter profiles.

### Cell Separation

γδ T cells were purified by negative selection from the peripheral lymph nodes (pLNs; inguinal, axillary, brachial and cervical) of CD28^+/+^ γδTCR Tg, CD28^−/−^ γδTCR Tg and TCRα^−/−^ mice using the MACS® magnetic bead separation system (Miltenyi Biotech, Auburn, CA, USA). First, cells were stained for 10 minutes at 4°C with a panel of FITC-labeled mAbs containing anti-CD19, anti-TCRβ, anti-CD4, anti-CD8, anti-CD11b, anti-IA^b^ and anti-DX5 mAbs. Next, cells were washed, incubated with anti-FITC MACS beads for 15 minutes at 4°C, and then separated on an autoMACS cell separator, according to manufacturer’s directions. Typically, the purity of the resulting DN γδ T cell populations from γδTCR Tg mice was ≥99%, and that of DN γδ T cells from TCRα^−/−^ mice was ≥85%.

αβ T cells were purified from the pLNs of CD28^+/+^ mice using the MACS® magnetic bead separation system described above, except that the following panel of FITC-conjugated antibodies was used: anti-CD19, anti-TCRγδ, anti-CD44, anti-CD11b, anti-IA^b^ and anti-DX5 mAbs. The purity of the resulting cell αβ T cell population was typically ≥98%.

### *In Vitro* Stimulation of γδ T Cells

Purified γδ T cells were resuspended in RPMI 1640 supplemented with non-essential amino acids, L-glutamine, HEPES, sodium pyruvate, and penicillin/streptomycin (all from Invitrogen) in addition to 10% FBS (Mediatech, Inc., Manassas, VA, USA), plated onto 5 µg/ml of immobilized hamster isotype control, 5 µg/ml of immobilized anti-CD3 mAb or 5 µg/ml each of immobilized anti-CD3 and anti-CD28 mAbs, and then cultured for 16 hours at 37°C. Cells were treated with Brefeldin A and Monensin (eBioscience) 5 hours prior to fixation, permeabilization, and intracellular staining with mAbs against IL-17A and IFNγ.

### Bacteria and Bacterial Infection of Mice

*Listeria monocytogenes* (Lm; strain 10403S) and Lm expressing the stable recombinant protein Lm ActA-Ub-acidic polymerase (PA)-SIINFEKL-FLAG [22] were grown as previously described [23]. Briefly, bacteria were grown overnight in 1 mL Brain Heart Infusion (BHI) broth (Teknova, Hollister, CA, USA) supplemented with 200 µg/mL streptomycin (Fisher Scientific, Pittsburgh, PA, USA). Overnight cultures were used at a 1∶10 (vol/vol) dilution to inoculate fresh BHI broth supplemented with 200 µg/mL streptomycin. Bacteria were incubated in an orbital shaker for 1–2 hours at 37°C to mid-log phase, and the bacterial concentration was determined by measuring absorbance at 600 nm. Mice were infected with 3×10^4^ CFU Lm by i.p. inoculation.

To assess γδ T cell effector function following Lm infection, spleens were harvested on days 1 and 5 post infection and processed into single cell suspensions in RPMI 1640, supplemented with non-essential amino acids, L-glutamine, HEPES, sodium pyruvate, penicillin/streptomycin, and 10% FBS. 3×10^6^ splenocytes were then plated onto 1 or 5 µg/ml of immobilized anti-TCRγδ mAb (UC7-13D5) or 5 µg/ml of immobilized hamster isotype control and cultured at 37°C for 6 hours in the presence of Brefeldin A and Monensin before intracellular staining with mAbs against IL-17A and IFNγ.

To assess CD8^+^ αβ T cell effector function following Lm infection, spleens were harvested on day 7 and processed into single cell suspensions in supplemented RPMI 1640 as above. 2.5×10^6^ splenocytes were cultured with 1 µM SIINFEKL peptide or 1 µM of an irrelevant peptide (both from Genscript, Piscataway, NJ, USA) at 37°C for 5 hours in the presence of Brefeldin A before intracellular staining with a mAb against IFNγ.

For bacterial colony counts, livers were harvested at day 5 post infection and then processed into a single cell suspension using the gentleMACS Dissociator (Miltenyi). The manufacturer-provided protocol for preparation of single-cell suspensions from mouse liver (gentleMACS-Liver) was followed to step 11. Bacteria were liberated from eukaryotic cells by the addition of 0.1% Triton-X 100 at a 1∶1 (vol/vol) dilution. Ten-fold serial dilutions of the lysates were plated onto BHI plates supplemented with 200 µg/mL streptomycin. Bacteria colonies were enumerated the following day.

### Glucose Uptake

Purified αβ and γδ T cells were cultured at 37°C in glucose-free RPMI (Invitrogen) supplemented with 30 µM 2-(N-(7-Nitrobenz-2-oxa-1,3-diazol-4-yl) Amino)-2-Deoxyglucose (2-NBDG) (Invitrogen) at 5×10^5^ cells per well, in a 48-well plate, in the presence or absence of 1 µg/mL plate-bound anti-CD3 mAb. T cells were harvested from the plate at various time points, and the amount of 2-NBDG taken up by the cells was measured by flow cytometry.

### Measuring Glucose-dependent Proliferation and Cytokine Production

Purified γδ T cells were labeled with 5-carboxyfluorescein diacetate succinimidl ester (CFSE), according to the manufacturer’s instructions (eBioscience). Cells were cultured in glucose-free RPMI supplemented with non-essential amino acids, L-glutamine, HEPES, sodium pyruvate, penicillin/streptomycin, 10% dialyzed FBS (Invitrogen) and various concentrations of D-glucose at 3×10^5^ cells per well, in a 48-well plate, in the presence of 0.2 or 1 µg/mL plate-bound anti-CD3 mAb. After 48 hours at 37°C, cells were harvested from the plate and cellular proliferation was analyzed by flow cytometry. Supernatants were also collected and analyzed by ELISA for the presence of IL-2, IFNγ, and IL-17A, according to the manufacturer’s (eBioscience) instructions.

### Statistical Analysis

Data are presented as mean ± SEM. Student’s t-test was used for all statistical comparisons (Graph Pad Prism or Microsoft Excel software). Only *p* values less than or equal to 0.05 (i.e., statistically significant) are denoted.

## Results

### CD28 is Differentially Expressed on γδ T Cell Effector Subsets

**Figure 1:**
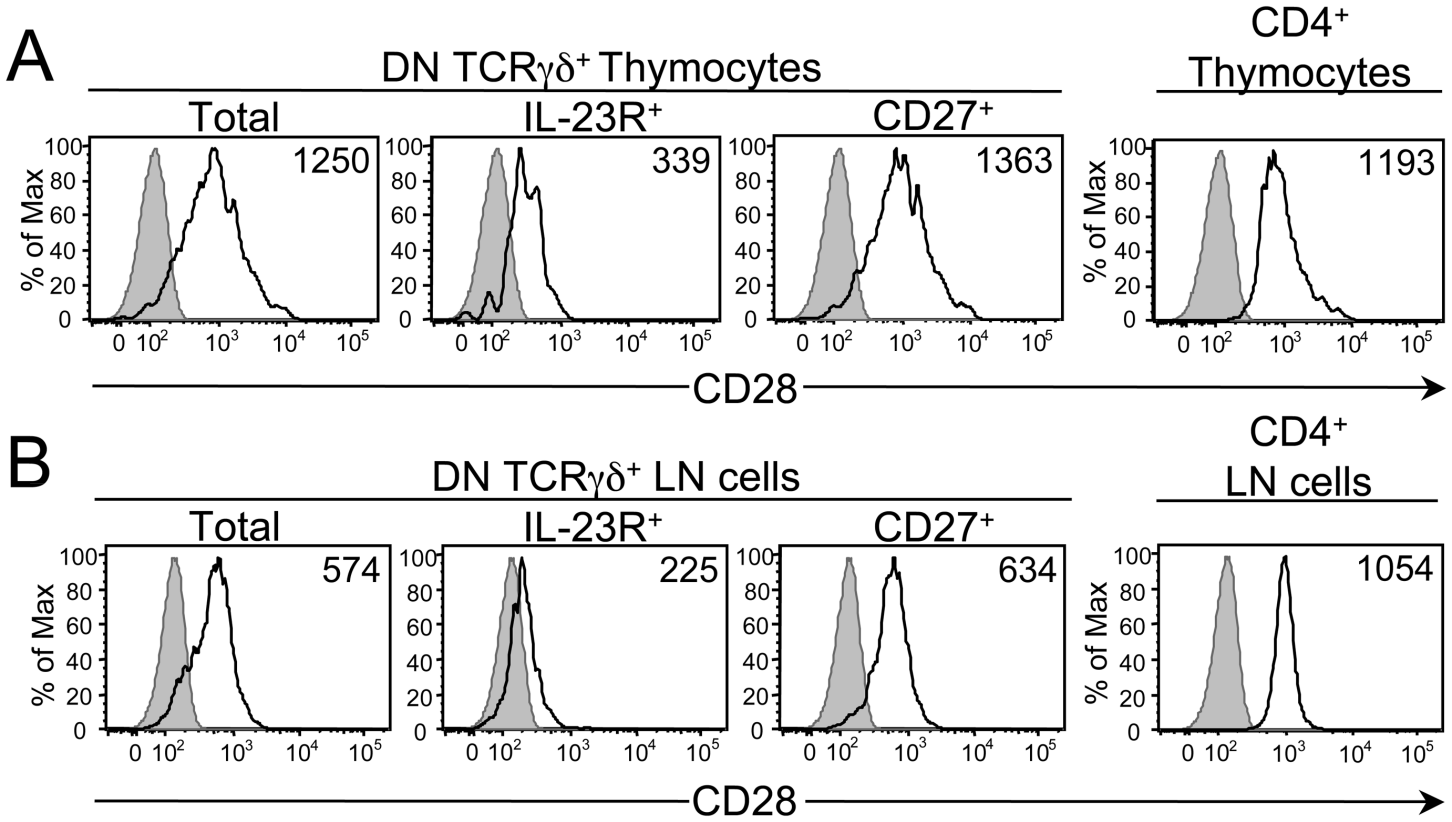
Comparison of CD28 expression levels on γδ T cell subsets in the thymus and periphery. Analysis of CD28 expression on various gated subsets in the thymus (**A**) and pLNs (**B**) of CD28^+/+^ (i.e., IL-23R^gfp/+^) mice. Black histograms show representative staining of CD28 on total, IL-23R^+^ (GFP^+^) and CD27^+^ DN TCRγδ^+^ subsets as well as on CD4^+^ TCRαβ^+^ subsets. Staining of thymocytes (**A**) and pLN cells (**B**) from CD28^−/−^ mice are shown as negative controls (shaded histograms). Numbers in the plots represent the mean fluorescent intensity (MFI) of CD28 expression. Data are representative of six mice in three independent experiments.

Our re-evaluation of the role of CD28 in γδ T cell development and differentiation began with comparing CD28 expression levels on the recently identified γδ-IFNγ and γδ-17 effector subsets. To optimize the detection of CD28 expression on the cell surface, we stained cells with a PE-conjugated anti-CD28 mAb and ran them on a flow cytometer fitted with a 568 nm laser, which excites PE significantly better than the conventional 488 nm laser [24]. γδ-IFNγ and γδ-17 effector subsets can be identified in the thymus and periphery by their expression of different surface markers. All γδ-IFNγ cells express the TNFR family member CD27, with a subset of them expressing CD122 [18], [19]. γδ-17 cells express neither CD27 nor CD122 [18], [19] but instead express IL-23R and the chemokine receptor CCR6 [20], [25]–[27]. Using CD27 and IL-23R to detect γδ-IFNγ and γδ-17 cells, respectively, we found that CD28 expression levels were 2 to 3-fold higher on γδ-IFNγ cells than on γδ-17 cells, regardless of whether the γδ T cells were from C57BL/6 or Vγ6/Vδ1 γδTCR Tg mice (Figure 1 and Figure S1). Since the CD27^+^ IFNγ-producing γδ T cell effector subset consists of both CD122^–^ and CD122^+^ cells, we analyzed each subpopulation and found that their CD28 expression levels were comparable (data not shown). In addition, we noted that γδ-IFNγ and γδ-17 cells in the thymus expressed CD28 at considerably higher levels than their peripheral counterparts. This is in contrast to CD4^+^ thymocytes and CD4^+^ LN cells, which were found to express equivalent levels of CD28 (Figure 1A and B). Taken together, these data demonstrate that CD28 is differentially expressed between γδ-17 and γδ-IFNγ cells and, as these cells leave the thymus and enter the periphery, they downregulate their expression of CD28.

### CD28 Deficiency Results in Reduced Numbers of γδ Lineage Cells

**Figure 2:**
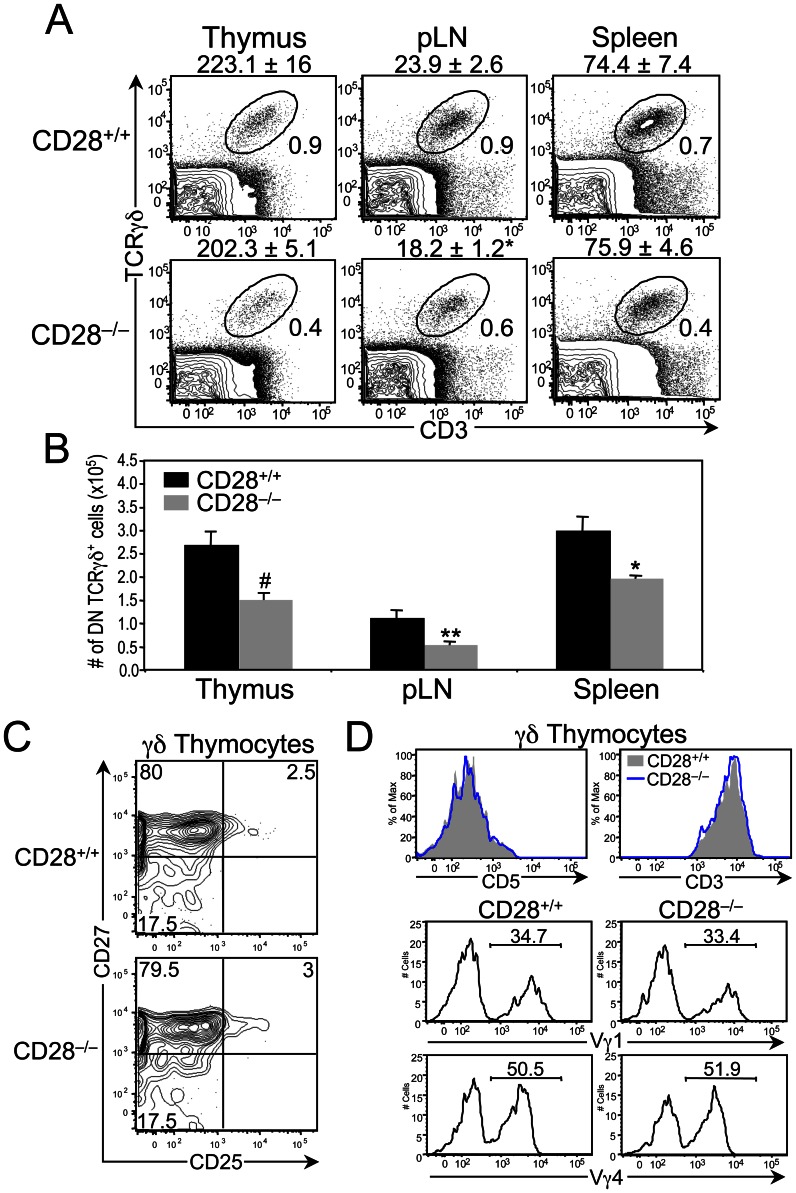
Effect of CD28 deficiency on γδ T cell development. (**A**) Phenotypic analysis of CD28^+/+^ and CD28^−/−^ mice. Dot plots show representative TCRγδ versus CD3 staining profiles on gated DN thymocytes, DN pLN cells and DN splenocytes. Numbers within the two-color plots represent the percentage of TCRγδ^+^ CD3^+^ cells in the gate. The mean cell number ± SEM for each tissue and genotype are displayed above the two-color plots. (**B**) Bars represent the mean number ± SEM of DN TCRγδ^+^ cells in the thymus, pLNs and spleen of CD28^+/+^ (n = 4 to 8) and CD28^−/−^ (n = 4 to 8) mice. *p≤0.05, **p≤0.01 and *#p≤0.001*. (C) Representative histograms comparing CD3 and CD5 levels on γδ (TCRγδ^+^) thymocytes from CD28^+/+^ (n = 8) and CD28^−/−^ (n = 8) mice. (D) Representative histograms comparing the percentages of Vγ1^+^ and Vγ4^+^ γδ thymocytes from CD28^+/+^ (n = 8) and CD28^−/−^ (n = 8) mice.

Given the relatively high expression levels of CD28 on DN γδ thymocytes, we next sought to determine whether CD28 is required for γδ T cell development by comparing the ability of CD28^+/+^ and CD28^−/−^ mice to generate γδ lineage cells. Significant decreases were observed in both the percentage and number of TCRγδ^+^ cells in the thymus, spleen, and pLNs of CD28^−/−^ mice compared to CD28^+/+^ mice (Figure 2A and B). The reduction in γδ T cell numbers was not due to a partial block in early γδ T cell development, as the percentage of CD25^+^ CD27^+^ γδ thymocytes in CD28^+/+^ and CD28^−/−^ mice was equivalent (Figure 2C). Furthermore, phenotypic analysis of the γδ lineage cells from CD28^−/−^ mice revealed no appreciable differences in CD5 and γδTCR surface levels, or in Vγ usage, relative to the γδ lineage cells from CD28^+/+^ mice (Figure 2D), demonstrating that γδTCR signal strength and γδTCR repertoire selection were not altered by the lack of CD28 costimulation.

**Figure 3:**
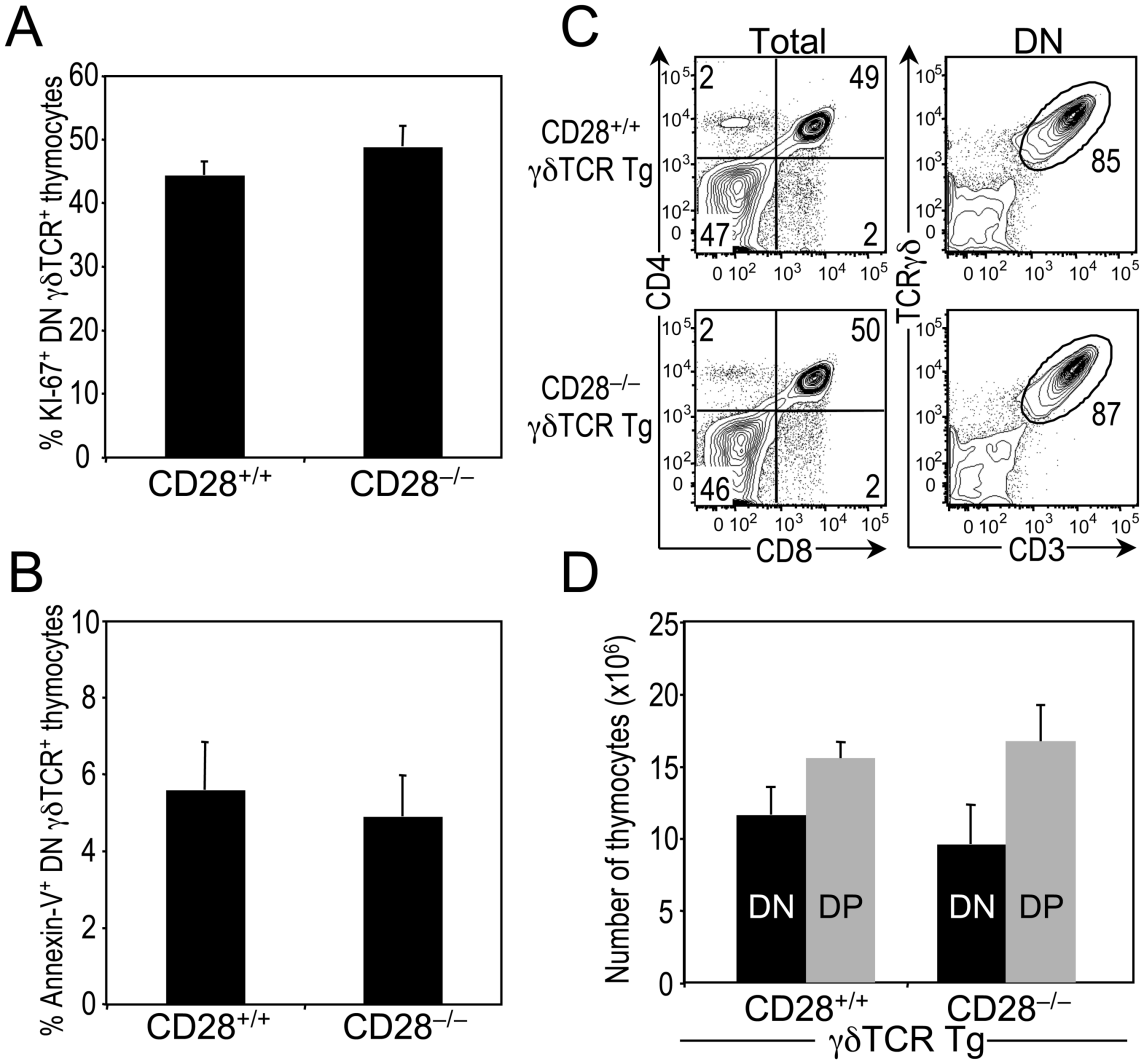
Effects of CD28 deficiency on γδ thymocyte proliferation, cell death and lineage commitment. (**A**) Bars represent the mean ± SEM of the percentage of Ki-67^+^ DN TCRγδ^+^ thymocytes from CD28^+/+^ (n = 8) and CD28^−/−^ (n = 8) mice. (**B**) Bars represent the mean percent ± SEM of Annexin V^+^ DN TCRγδ^+^ thymocytes from CD28^+/+^ (n = 4) and CD28^−/−^ (n = 4) mice. (**C**) Phenotypic analysis of CD28^+/+^ γδTCR Tg and CD28^−/−^ γδTCR Tg mice. Dot plots show representative CD4 versus CD8 staining profiles on total thymocytes. Numbers in quadrants of the two-color plots represent the percentage of cells in each quadrant. Adjacent dot plots show representative TCRγδ versus CD3 staining on gated DN thymocytes. Numbers represent percentage of cells in each gate. Data are representative of three independent experiments. (**D**) Mean number ± SEM of DN (DN TCRγδ^+^; γδ lineage) and DP (αβ lineage) thymocytes in CD28^+/+^ γδTCR Tg and CD28^−/−^ γδTCR Tg mice. Data represent seven mice per genotype.

There are several possible explanations for the loss of γδ T cells in CD28^−/−^ mice. The first is that CD28 signaling is required for the optimal proliferation and/or survival of DN γδ thymocytes. To test this possibility, we used flow cytometric analysis to compare the percentages of CD28^+/+^ and CD28^−/−^ DN γδ thymocytes expressing the Ki-67 antigen, a marker for actively cycling cells, and staining positive for Annexin-V, a marker of apoptosis. There were no differences in the proportions of Ki-67^+^ or Annexin-V^+^ DN γδ thymocytes between CD28^+/+^ and CD28^−/−^ mice (Figure 3A and B), indicating that neither diminished cellular proliferation nor increased cell death can account for the reduced γδ T cell numbers in CD28^−/−^ mice.

Another possible explanation for the loss of γδ T cells in CD28^−/−^ mice is that CD28 signaling plays a role in commitment to the γδ lineage. To investigate this possibility, we used a γδTCR Tg mouse model in which the αβ/γδ lineage decision is mediated by the γδTCR and in which alterations in αβ/γδ lineage choice can be detected by enumerating the number of γδ lineage (DN TCRγδ^+^) and αβ lineage (CD4^+^ CD8^+^; DP) thymocytes [28]. In the thymus of γδTCR Tg CD28^+/+^ and γδTCR Tg CD28^−/−^ mice, we found no significant differences in the numbers of αβ and γδ lineage cells (Figure 3C and D), demonstrating that, in the absence of CD28, there is no inherent defect in γδ lineage commitment.

### CD28 Deficiency Affects the Early Stages of T Cell Development

**Figure 4:**
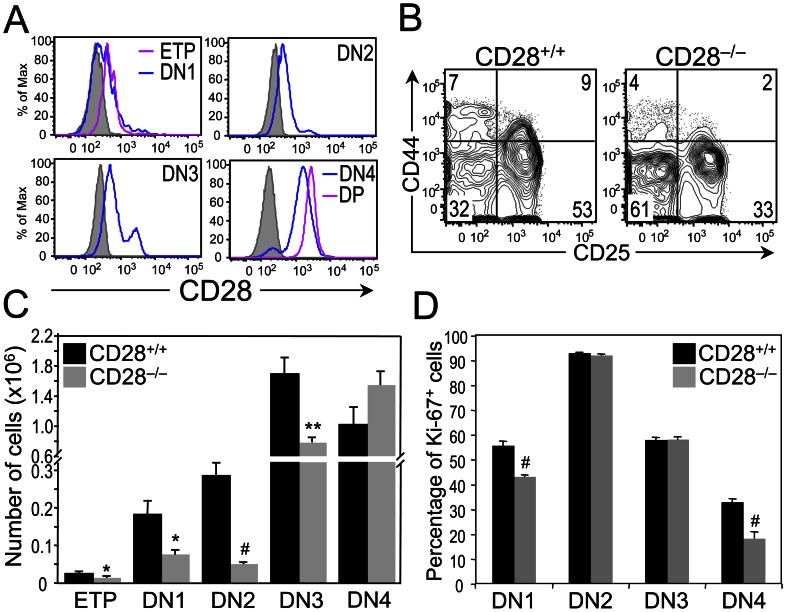
Effects of CD28 deficiency on early T cell development. (**A**) Histograms showing staining of CD28 on gated ETP [lineage-negative (lin^–^) CD25^–^ CD44^+^ CD117^+^], DN1 (lin^–^ CD25^–^ CD44^+^), DN2 (lin^–^ CD25^+^ CD44^+^), DN3 (lin^–^ CD25^+^ CD44^–^) and DN4 (lin^–^ CD25^–^ CD44^–^) and DP (CD4+ CD8+) subsets, where lin^–^ is defined as CD4^–^ CD8^–^ CD11b^–^ CD19^–^ CD49b^–^ TCRβ^–^ TCRγδ^–^ NK1.1^–^ IA^b–^. Staining of thymocytes from CD28^−/−^ mice are shown as negative controls (shaded histograms). Data are representative of seven mice. (**B**) Dot plots show representative CD44 versus CD25 staining on gated lin^–^ thymocytes from CD28^+/+^ (n = 4) and CD28^−/−^ (n = 4) mice. Numbers in quadrants represent percentage of cells in each quadrant. (**C**) Bars represent the mean cell number ± SEM of ETPs, DN1 DN2, DN3, and DN4 thymocytes from CD28^+/+^ (n = 4) and CD28^−/−^ (n = 4) mice. (**D**) Bars represent the mean percentage of Ki-67^+^ cells ± SEM of gated DN thymocyte subsets from CD28^+/+^ (n = 4) and CD28^−/−^ (n = 4) mice. *p ≤ 0.05, **p ≤ 0.01, #*p* ≤ 0.001.

Since CD28 signaling has no apparent role in γδ lineage commitment or in γδ thymocyte proliferation and survival, we next examined the possibility that CD28 signaling is required prior to the expression of the γδTCR, for the proliferation and/or survival of the thymic progenitors that give rise to γδ T cells. Consistent with a previous report [29], we detected CD28 expression on the surface of all immature DN thymocytes, with CD28 surface levels increasing as thymocytes transition through the DN1 (lineage^–^ CD44^+^ CD25^–^), DN2 (lineage^–^ CD44^+^ CD25^+^) and DN3 (lineage^ –^ CD44^–^ CD25^+^) stages to the DN4 stage (lineage^ –^ CD44^–^ CD25^–^) (Figure 4A). To determine whether CD28 expression is required for the progression of thymocytes through the DN stages, we compared the distribution, proliferative status and viability of DN subsets in CD28^+/+^ and CD28^−/−^ mice. Notably, we found that the percentages and numbers of DN1 [including the early thymic progenitor (ETP) subset], DN2 and DN3 thymocytes were decreased, while those of DN4 thymocytes were equivalent, in CD28^−/−^ mice compared to CD28^+/+^ mice (Figure 4B and C). Comparison of the proliferative status of the DN subsets in CD28^+/+^ and CD28^−/−^ thymi revealed that the percentages of proliferating thymocytes in the DN1 and DN4 subsets, but not the DN2 and DN3 subsets, were significantly reduced in the absence of CD28 (Figure 4D). Moreover, only the DN4 subset in CD28^−/−^ mice exhibited increased cell death, as evidenced by the higher percentage of Annexin V^+^ DN4 thymocytes in CD28-deficient mice than in CD28-sufficient mice (Table 1). Taken together, these data indicate that proliferation of DN1 thymocytes is impaired in CD28^−/−^ mice and suggest that CD28 signaling regulates the size of the thymic progenitor pool. Importantly, considering that thymocytes committing to the γδ lineage undergo limited proliferation [30], it follows that CD28^−/−^ mice, which have fewer thymic progenitors than CD28^+/+^ mice, generate fewer γδ T cells.

**Table 1:** Effect of CD28-deficiency on survival of DN thymocyte subsets.

Genotype	% Annexin V^+^ DN1	% Annexin V^+^DN2	% Annexin V^+^DN3	% Annexin V^+^DN4
CD28^+/+^	36.0±3.8	3.5±0.9	3.6±0.1	9.6±4.7
CD28^−/−^	38.9±10.5	3.0±0.7	3.9±0.4	33.8±2.7**

** p≤0.01; n = 4 mice per genotype.

### CD28 Deficiency Does Not Affect γδ T Cell Effector Fate Commitment or Function

**Figure 5:**
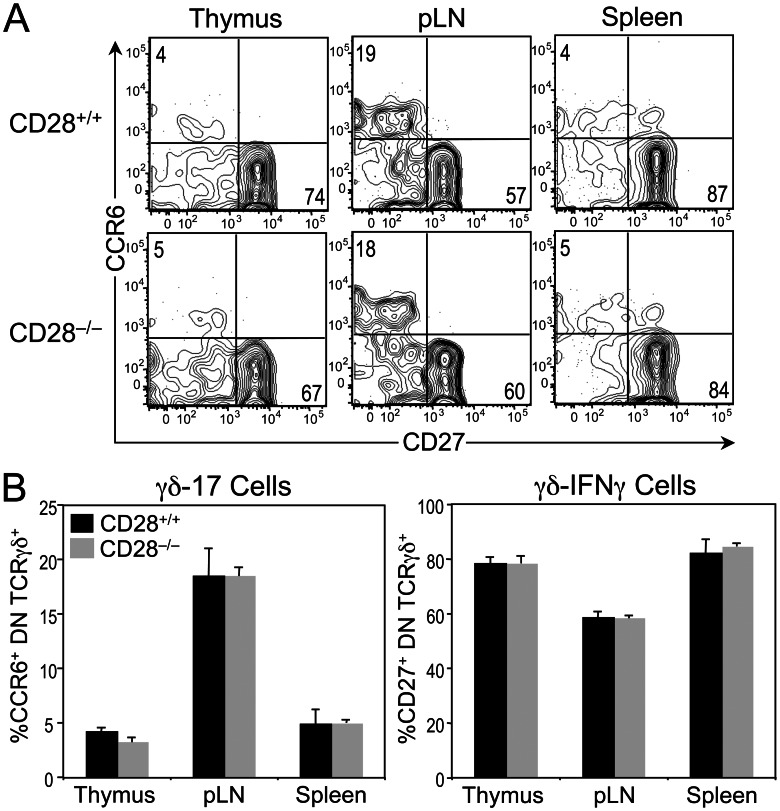
Effect of CD28 deficiency on the acquisition of γδ effector fates in the thymus. (A) Dot plots represent CCR6 versus CD27 staining profiles on gated DN TCRγδ^+^ cells from thymus, pLN and spleen of CD28^+/+^ (n = 7) and CD28^−/−^ (n = 7) mice. Numbers in quadrants represent the percentage of cells in that quadrant. (**B**) Right panel: bars represent the mean percentage ± SEM of γδ-17 (CCR6^+^ CD27^–^ DN TCRγδ^+^) cells. Left panel: bars represent the mean percentage ± SEM of γδ-IFNγ (CCR6^–^ CD27^+^ DN TCRγδ^+^) cells.

Because of the difference in CD28 expression levels on γδ-17 and γδ-IFNγ cells, we sought to determine whether these effector lineages had a differential requirement for CD28 in their development or differentiation. To test whether CD28 signaling is required to generate γδ-17 or γδ-IFNγ cells, we measured the frequency of CCR6^+^ CD27^–^ (γδ-17) and CCR6^–^ CD27^+^ (γδ-IFNγ) DN TCRγδ^+^ cells in CD28-sufficient and CD28-deficient mice. Although there were fewer γδ lineage cells in CD28^−/−^ mice (Figure 2B), we observed no differences in the distribution of CCR6^+^ CD27^–^ and CCR6^–^ CD27^+^ cells in the thymus, spleen and pLNs between CD28^+/+^ and CD28^−/−^ mice (Figure 5A and B). These findings indicate that CD28 signaling does not play a role in commitment to either effector fate.

**Figure 6:**
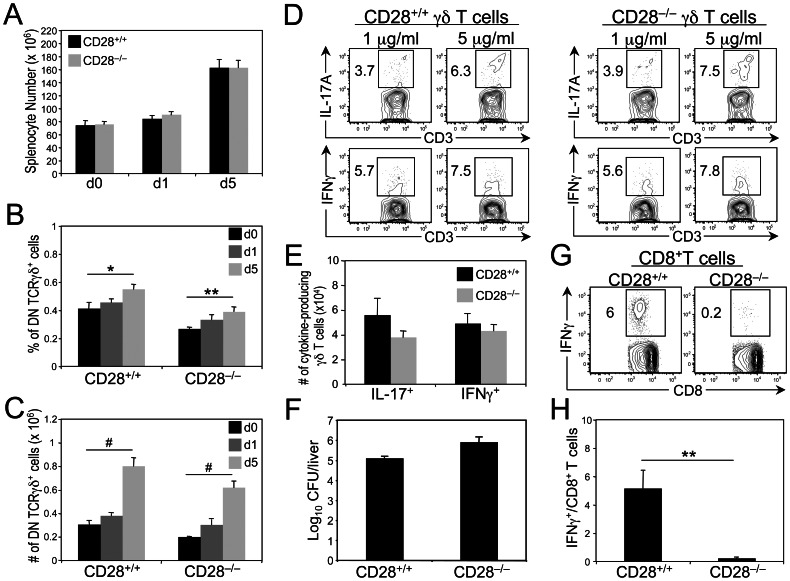
Role of CD28 costimulation in *in vivo* γδ T cell activation and differentiation. CD28^+/+^ and CD28^−/−^ mice were infected i.p. with 3×10^4^ CFU Lm and sacrificed on day 1 and day 5 post infection. (**A**) Bars represent the mean number ± SEM of cells in the spleens of CD28^+/+^ and CD28^−/−^ mice at days 0, 1 and 5 post infection. Data represent four (day 0), five (day 1) and thirteen (day 5) mice per genotype. (B) Bars represent the mean percentage ± SEM of DN γδ T cells in the spleens of CD28^+/+^ and CD28^−/−^ mice at days 0, 1 and 5 post infection. Data represent four (day 0), five (day 1) and thirteen (day 5) mice per genotype (**C**) Bars represent the mean number ± SEM of DN γδ T cells in the spleens of CD28^+/+^ and CD28^−/−^ mice at days 0, 1 and 5 post infection. Data represent four (day 0), five (day 1) and thirteen (day 5) mice per genotype. (**D**) Dot plots show representative i.c. staining for IFNγ and IL-17A on gated TCRγδ^+^ cells of CD28^+/+^ and CD28^−/−^ mice on day 5 post infection, *in vitro* re-stimulated with either 1 or 5 µg/ml anti-TCRγδ mAb. Numbers in gate represent percentage of cells in that gate. (**E**) Bars represent the mean cell number ± SEM of IFNγ- and IL-17A-producing TCRγδ^+^ cells in the spleens of Lm-infected CD28^+/+^ (n = 13) and CD28^−/−^ (n = 13) mice on day 5 post infection. (**F**) Enumeration of the bacterial counts (i.e, CFU) in the liver of CD28^+/+^ and CD28^−/−^ mice on day 5 post infection. Data represent 4 mice per genotype. (**G, H**) CD28^+/+^ and CD28^−/−^ mice were infected i.p. with 3×10^4^ CFU Lm expressing the stable recombinant protein Lm ActA-Ub-PA-SIINFEKL-FLAG and sacrificed on day 7 post infection. (**G**) Dot plots show representative i.c. staining for IFNγ on gated CD8^+^ αβ T cells of CD28^+/+^ and CD28^−/−^ mice on day 7 post infection, *in vitro* re-stimulated with 1 µM SIINFEKL peptide. (**H**) Bars represent the mean percentage ± SEM of IFNγ-producing SIINFEKL-specific CD8^+^ αβ T cells in the spleens of Lm-infected CD28^+/+^ (n = 5) and CD28^−/−^ (n = 5) mice on day 7 post infection. *p ≤ 0.05, **p ≤ 0.01, #*p* ≤ 0.001.

We next investigated whether CD28 costimulation is required for the differentiation of γδ-17 and γδ-IFNγ cells into cytokine-producing effectors. We and others have previously demonstrated that *in vitro* activation of γδ T cells through TCR stimulation alone is sufficient to induce not only robust proliferation but also IL-17 and IFNγ production [18], [31]–[35]. To determine whether cytokine production by γδ T cells is enhanced by γδTCR and CD28 co-engagement, we stimulated purified γδ T cells, from both γδTCR Tg and TCRα^−/−^ mice, with anti-CD3 mAb in the presence or absence of anti-CD28 mAb. Although IFNγ production was modestly enhanced, no change in IL-17A production was observed following TCR/CD28 co-engagement (Figure S2A). To determine whether CD28 costimulation is required for *in vivo* γδ T cell effector functions, we infected CD28^+/+^ and CD28^−/−^ mice with *Listeria monocytogenes*, a model pathogen that not only induces IL-17A- and IFNγ-producing γδ T cells [36], [37], but also upregulates the expression of the CD28 ligands, B7.1 and B7.2, on antigen presenting cells early in the course of infection [38–40; data not shown]. Leading up to day 5 post infection, the peak of the γδ T cell response [36], [37], [40], [41], we noted no difference in the total number of splenocytes in CD28^+/+^ and CD28^−/−^ mice (Figure 6A). Furthermore, on days 1 and 5 post infection, equivalent fold increases in the percentage and number of splenic γδ T cells were observed in both genotypes (Figure 6B and C), indicating that CD28 deficiency has no effect on the expansion and/or recruitment of γδ T cells in response to Lm. When we compared the ability of CD28^+/+^ and CD28^−/−^ γδ T cells to differentiate into cytokine-producing cells, we observed similar percentages and numbers of IL-17A^+^ and IFNγ^+^ γδ T cells in both genotypes, regardless of the duration of infection (Figure S2B) or the dose of anti-TCRγδ mAb used to re-stimulate γδ T cell effectors *in vitro* (Figure 6D and E). Because γδ-17 and γδ-IFNγ cells play a critical role in mediating bacterial clearance [36], [37], [40], [41], we also enumerated bacterial CFU in the liver on day 5 post infection. Consistent with equivalent numbers of IL-17A^+^ and IFNγ^+^ γδ T cells in infected CD28^+/+^ and CD28^−/−^ mice, we observed no difference in the bacterial burden of CD28^+/+^ and CD28^−/−^ livers (Figure 6F). Together, these findings demonstrate that neither γδ-17 nor γδ-IFNγ cells require *in vivo* CD28 costimulation during Lm infection to differentiate into cytokine-producing effectors.

It has been previously reported that Lm-specific CD8^+^ αβ T cell effectors require CD28 costimulation [38], [42]. To confirm this finding in our system, we infected CD28^+/+^ and CD28^−/−^ mice with recombinant Lm expressing a protein containing SIINFEKL, an ovalbumin-derived MHC Class I-restricted determinant. On day 7 post infection, we quantified SIINFEKL-specific CD8^+^ αβ T cells by intracellular IFNγ staining. In agreement with the previous reports, we observed dramatically fewer IFNγ^+^ CD8^+^ αβ T cells in CD28^−/−^ mice than in CD28^+/+^ mice (Figure 6G and H). Collectively, these findings highlight major differences in the molecular requirements for the generation of αβ and γδ T cell effectors during the course of Lm infection.

### Differences in Glucose Uptake Between Resting αβ and γδ T Cells

**Figure 7:**
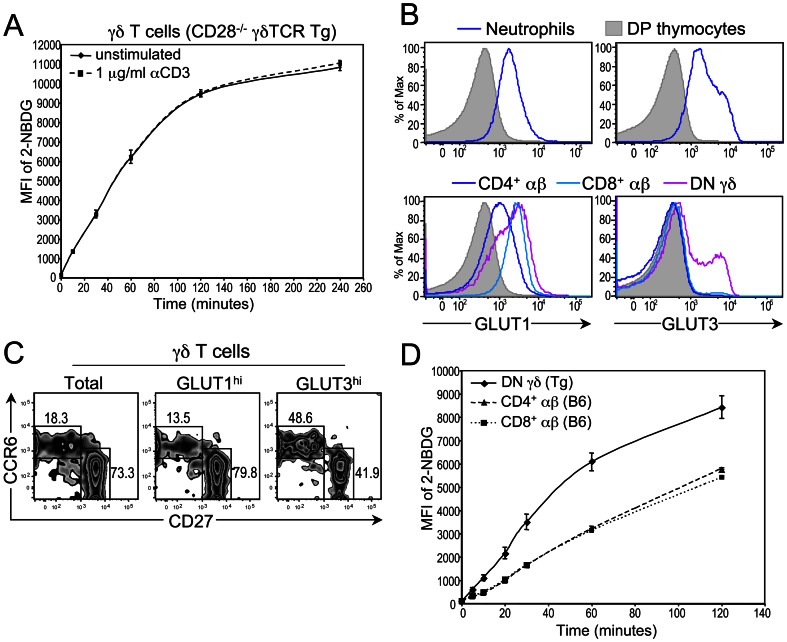
Glucose uptake by, and expression of GLUT isoforms on, γδ T cells. (**A**) Purified γδ T cells from either CD28^+/+^ γδTCR Tg or CD28^−/−^ γδTCR Tg mice were cultured in glucose-free medium supplemented with 30 µM 2-NBDG in the presence or absence of 1 µg/mL plate-bound anti-CD3 mAb. γδ T cells were harvested from the plate at various time points and 2-NBDG uptake was measured by flow cytometric analysis. Graph shows MFI of 2-NBDG as a function of time for unstimulated (black line) and stimulated (dashed line) γδ T cells. Data are representative of three independent experiments; the data from the CD28^−/−^ γδ T cells are shown. (**B**) Top panel: GLUT1 and GLUT3 expression levels on neutrophils (CD11b^+^ Ly-6G^+^; blue histograms) and DP thymocytes (shaded histograms) from CD28^+/+^ mice. Bottom panel: GLUT1 and GLUT3 expression levels on CD4^+^ CD44^–^ αβ T cells, CD8^+^ CD44^–^ αβ T cells, and DN γδ T cells. Staining on DP thymocytes (shaded histogram) is also shown as a negative control. Data are representative of three independent experiments, using at least 9 CD28^+/+^ mice. (**C**) Distribution of γδ-17 (CCR6^+^ CD27^–^) and γδ-IFNγ (CCR6^–^ CD27^+^) within all γδ T cells and within the GLUT1^hi^ and GLUT3^hi^ γδ T cell subsets. (**D**) Comparison of glucose uptake between unstimulated αβ and γδ T cells. Purified γδ T cells from CD28^+/+^ γδTCR Tg mice and purified αβ T cells from CD28^+/+^ mice were cultured in glucose-free medium supplemented with 30 µM 2-NBDG for various periods of time. 2-NBDG uptake as well as CD4 and CD8 expression was measured by flow cytometric analysis. Graph shows MFI of 2-NBDG as a function of time for γδ T cells (black line), CD4^+^ αβ T cells (dashed line) and CD8^+^ αβ T cells (dotted line). Data are representative of three independent experiments.

CD28 costimulation supports αβ T cell growth, proliferation and effector function by regulating glucose uptake and utilization [5], [6]. Accordingly, differences between αβ and γδ T cells in their regulation of glucose metabolism may explain the CD28 independence of γδ T cell activation, expansion and differentiation during Lm infection. To test this, we first assessed the ability of γδ T cells from both CD28^+/+^ and CD28^−/−^ mice to take up 2-NBDG, a fluorescent glucose derivative, in the presence or absence of CD3 engagement. We used γδTCR Tg mice for this assay, as large numbers of DN γδ T cells can be purified from these mice by negative selection [31], [33], [34], which eliminates any concern that purification with anti-TCRγδ mAbs results in γδ T cell activation. Remarkably, we observed no difference in the kinetics or magnitude of glucose uptake between resting and activated γδ T cells from either γδTCR Tg CD28^+/+^ or γδTCR Tg CD28^−/−^ mice (Figure 7A, D and data not shown), indicating that γδTCR signaling has minimum effect on glucose uptake. Because the ability of a cell to take up glucose is dependent on surface expression of glucose transporters (GLUTs), we next determined whether GLUT1 and GLUT3, the two isoforms expressed predominantly by leukocytes [43], [44], were differentially expressed by αβ and γδ T cells. To accomplish this, we developed a flow cytometric assay to detect surface expression of GLUT1 and GLUT3, using cells that are known to express high levels (e.g., neutrophils) and low levels (e.g., DP thymocytes) of the GLUT isoforms (Figure 7B) [43]–[46]. When we compared surface expression of these isoforms on peripheral αβ T cells and γδ T cells from CD28^+/+^ and CD28^−/−^ mice, we found that γδ T cells and CD8^+^ αβ T cells expressed higher surface levels of GLUT1 than CD4^+^ αβ T cells (Figure 7B and S3A). GLUT3 expression, on the other hand, was detected only on γδ T cells (Figure 7B and S3A). Importantly, both γδ-17 and γδ-IFNγ cells were found within the GLUT1^hi^ and GLUT3^hi^ γδ T cell subsets, but a higher percentage of γδ-17 cells was contained within the GLUT3^hi^ subset than in the GLUT1^hi^ subset (Figure 7C). Interestingly, GLUT1 and GLUT3 expression levels were also expressed at relatively high levels on γδ thymocytes (Figure S3B), suggesting that GLUT1 and GLUT3 expression levels are induced in the thymus as part of a developmental program. Together, these data indicate that GLUT isoforms are differentially expressed between αβ and γδ T cells, with γδ T cells, on average, expressing higher surface levels of GLUT1 and GLUT3 than αβ T cells. This differential expression suggests that γδ T cells are better equipped to take up glucose than αβ T cells.

Because GLUT3 has a higher affinity for glucose, and a greater glucose transport capacity, than GLUT1 [46], we sought to determine whether there was a difference in the kinetics of glucose uptake between resting αβ and γδ T cells under low glucose conditions. Indeed, we found that γδ T cells take up 2-NBDG, when present at a low (0.03 mM) concentration, at a rate that is twice as fast as that of either CD4^+^ or CD8^+^ αβ T cells (Figure 7D), demonstrating that the GLUT3 on γδ T cells is functional and that γδ T cells have a higher basal rate of glucose uptake than αβ T cells.

### γδ T Cell Effector Function is Optimal over a Wide Range of Glucose Concentrations

**Figure 8:**
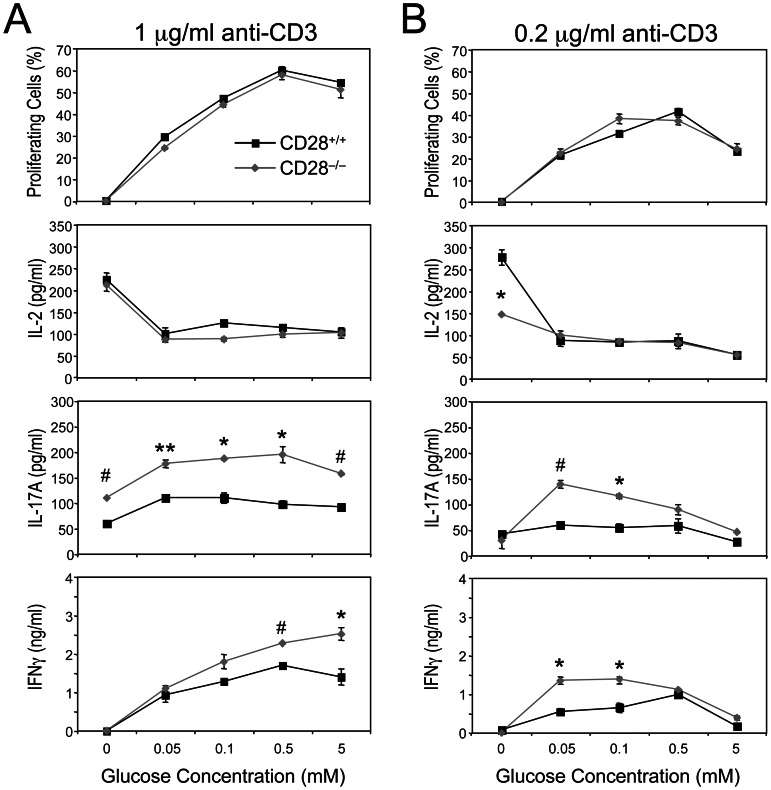
Effect of glucose concentration on γδ T cell proliferation and cytokine production. Purified γδ T cells from CD28^+/+^ γδTCR Tg and CD28^−/−^ γδTCR Tg mice were labeled with CFSE and then cultured in glucose-free medium or glucose-free medium supplemented with increasing concentrations of glucose in the presence of 1 µg/mL (**A**) or 0.2 µg/ml (**B**) of plate-bound anti-CD3 mAb. 48 h later, cells were harvested and their proliferative response was measured by flow cytometric analysis. Supernatants were also collected and cytokine production was measured by ELISA. Effect of glucose concentration on cellular proliferation, IL-2 production, IL-17A production, and IFNγ production. Data are representative of at least three mice per genotype. *p ≤ 0.05, **p ≤ 0.01, #*p* ≤ 0.001.

In addition to signals from the TCR and CD28, αβ T cells require relatively high glucose concentrations to proliferate and to produce cytokines (≥0.05 mM of glucose to produce IL-2 and ≥0.5 mM to proliferate and to produce IFNγ [6]). The ability of γδ T cells to produce cytokines during Lm infection independently of CD28 costimulation combined with their high GLUT1 and GLUT3 surface expression suggested that γδ T cells, when solely stimulated through the γδTCR, would exhibit effector functions over a wide range of glucose concentrations. To test this, we *in vitro* stimulated purified γδ T cells, from both γδTCR Tg CD28^+/+^ and γδTCR Tg CD28^−/−^ mice (Figure 8), in the presence of varying concentrations of glucose (0 to 5 mM), and then measured their ability to proliferate and produce various cytokines. When activated with a low but optimal dose of anti-CD3 mAb (1 µg/ml), we noted that γδ T cells from both genotypes proliferated in as little as 0.05 mM glucose and reached maximum proliferation at 0.5 mM glucose (Figure 8A). In addition, stimulated γδ T cells from both CD28^+/+^ and CD28^−/−^ mice produced low amounts of IL-17A, and high amounts of IL-2, in the absence of glucose (Figure 8A). However, while production of IL-2 decreased as glucose concentrations increased, production of both IL-17A and IFNγ improved under these same conditions, suggesting that ambient glucose concentrations regulate γδ T cell effector potential (Figure 8A). Surprisingly, we found that CD28-deficient γδ T cells produced, on average, more IL-17A and IFNγ than CD28-sufficient γδ T cells, irrespective of glucose concentration. These data suggest that CD28-B7 interactions restrain the production of IL-17A and IFNγ by γδ T cells.

It has been shown that γδ T cells require CD28 costimulation to proliferate when stimulated with a suboptimal dose of anti-CD3 mAb (<1 µg/ml) but not with an optimal dose of anti-CD3 mAb (≥1 µg/ml) [47], which is consistent with high TCR occupancy or a “strong” signal 1 compensating for the lack of CD28 costimulation [48]. However, as the proliferation assays were performed in RPMI 1640 [47], which contains glucose at a concentration of 11 mM, twice the glucose concentration found in blood [49]–[51], we next sought to determine whether low glucose concentrations altered the ability of γδ T cells to proliferate and produce cytokines following stimulation with a “suboptimal” dose (0.2 µg/ml) of anti-CD3 mAb. Strikingly, we found that the magnitude of the proliferative and cytokine responses of activated CD28-deficient and CD28-sufficient γδ T cells was higher when glucose concentrations were ≤0.5 mM than when the concentration was 5 mM (Figure 8B). These data indicate that ambient glucose concentrations play an important role in regulating γδ T cell activation, proliferation, and effector function.

## Discussion

To determine whether γδ T cells require CD28 signaling in the thymus during effector fate acquisition, or in the periphery during effector cell differentiation, we re-evaluated the effects of CD28-deficiency on γδ T cell development and function. We report that CD28 costimulation is not required for commitment to the γδ lineage, proliferation and survival of γδ thymocytes, nor adoption of the γδ-17 and γδ-IFNγ effector fates. Furthermore, in both *in vitro* and *in vivo* functional assays, we demonstrated that CD28 costimulation is dispensable for the generation of both IL-17^+^ and IFNγ^+^ γδ T cell effectors. To understand why γδ T cells are less dependent on CD28 costimulation than αβ T cells for their activation and differentiation, we investigated whether γδ T cells are endowed with properties that bypass the requirement for CD28 costimulation. Indeed, when we measured glucose uptake and utilization by γδ T cells, we noted significant differences between αβ and γδ T cells in their surface levels of GLUT1 and GLUT3 and in their threshold concentration of glucose required for optimal effector cell function. Together, these data underscore significant differences in the molecular requirements for the generation of αβ and γδ T cell effectors.

The re-evaluation of the role of CD28 in γδ T cell development revealed that CD28 signaling regulates the size of the thymocyte progenitor pool by controlling the proliferative ability of DN1 thymocytes. CD28 is expressed on the most immature thymocyte populations, including ETPs, whereas its ligands, B7.1 and B7.2, are expressed in the medulla and, to a lesser extent, the cortex [29], [52]. Once progenitors colonize the thymus through the vasculature located at the cortico-medullary junction, they traverse the cortex, initiating a T cell developmental program as they migrate toward the subcapsule [53]. During their journey through the cortex, the immature DN thymocytes encounter and interact with B7-expressing cortical epithelial cells. According to our data, this interaction is necessary for optimal cellular proliferation by DN1 thymocytes. Although its mechanism of action is currently unknown, it is possible that CD28 signaling controls proliferation of DN1 thymocytes 1) by regulating expression of the components of the IL-7R, c-kit, Hedgehog and/or Notch signaling pathways, all of which play a role in DN thymocyte proliferation [54]–[58], or 2) by acting in concert with one or more of these signaling pathways to promote maximum proliferation. Regardless of the mechanism involved, it is important to note that because the CD28-B7 interaction occurs prior to the expression of a TCR isoform, these data provide evidence for CD28 delivering a unique signal during the early stages of T cell development.

Although CD28-B7 interactions play no appreciable role in γδ T cell development, maturation or effector fate specification in the thymus, they do have a role in early αβ T cell development, where they promote survival and cellular proliferation of DN4 thymocytes [52)]. Our data showing that CD28^−/−^ DN4 thymocytes undergo more apoptosis and less proliferation than their wild-type counterparts are consistent with these findings. However, it is unclear how DN4 thymocyte numbers remain equivalent in CD28^+/+^ and CD28^−/−^ mice, in the face of the significant effects of CD28-deficiency on DN4 thymocyte survival and proliferation. Possible explanations include an accelerated transition time between the DN3 and DN4 stages [52] and a slower transition time between the DN4 and DP stages [59].

αβ T cells are dependent upon CD28 costimulation to upregulate expression of GLUTs on their cell surface as well as the enzymes involved in the glycolytic pathway [5], [6], [60]. This is not the case for γδ T cells, as both CD28^+/+^ and CD28^−/−^ γδ T cells express relatively high levels of GLUT1 and GLUT3 and, when activated by anti-CD3 mAb alone, are able to proliferate and secrete IL-17 and IFNγ in relatively low glucose concentrations (0.05 mM). In fact, CD28^−/−^ γδ T cells produced significantly more IL-17A and IFNγ than CD28^+/+^ γδ T cells *in vitro*, suggesting that CD28 is a negative regulator of γδ T cell effector cytokine production. Given that γδ T cells and CD8^+^ αβ T cells express equivalent levels of GLUT1 but have different basal rates of glucose uptake, we propose that the ability of γδ T cell effectors to function in low glucose concentrations is due to their expression of GLUT3, which has a higher affinity for glucose (*K*_m_ = 1.4 mM) than GLUT1 (*K*_m_ = 6.9 mM) [46]. However, because GLUT3-deficiency is embryonic lethal [61], determining whether GLUT3 expression confers γδ T cells with the ability to differentiate into cytokine-producing effectors independently of CD28 costimulation during Lm infection awaits the generation of mice in which *Slc2a3*, the gene that encodes GLUT3, can be conditionally deleted in T cells.

It is interesting to note that the induction of GLUT1 and GLUT3 expression by γδ lineage cells occurs in the thymus, presumably as part of their developmental program. The relatively high expression of GLUT isoforms at an early stage in γδ T cell development raises the question as to how the expression of GLUT1 and GLUT3 is induced in γδ lineage cells. In light of previous studies showing that TCR stimulation, IL-7 signaling, and insulin signaling induce GLUT1 and GLUT3 expression on the surface of αβ T cells [43], [44], [62], [63], it is tempting to speculate that similar mechanisms act in both γδ thymocytes to induce GLUT expression and in peripheral γδ T cells to maintain GLUT expression.

While we have found that γδ T cells do not require CD28 costimulation during Lm infection to generate γδ-17 and γδ-IFNγ effectors, a recent study reported that CD28 costimulation was required for the expansion of γδ-17 and γδ-IFNγ effectors during blood-stage Plasmodium infection [47]. One possible explanation for the discrepancy is that γδTCR occupancy is significantly higher during Lm infection than during Plasmodium infection, with the strong signal 1 compensating for the lack of CD28 costimulation. The idea that γδTCR occupancy is high during Lm infection is supported by the study of O’Brien and colleagues [64], which showed that the expression levels of the ligand for the Vγ6/Vδ1 TCR, the TCR expressed by one of the major γδ T cell subsets responding to Lm [36], are significantly increased on macrophages following Lm infection. Another possible explanation is that Lm infection, but not blood-stage Plasmodium infection, lowers glucose levels in the spleen to levels that permit the activation, expansion and differentiation of γδ T cell effectors in the absence of CD28 signaling. Interestingly, although both pathogens induce hypoglycemia in the host, significant reductions in blood glucose levels are observed as early as day 1 post Lm infection [49] but not until days 6 to 8 post blood-stage Plasmodium infection [50], [51]. Considering that γδ T cell effector function peaks on day 5 or earlier during the immune response [36], [37], [40], [41], [47] and that γδ T cell proliferation and cytokine production are measurably better in low glucose concentrations than in high glucose concentrations following stimulation with suboptimal doses of anti-CD3 mAb, this 5 to 7 day difference in the onset of hypoglycemia could have a great impact on the γδ T cell response.

In summary, we have shown that γδ T cells, unlike NKT and T_reg_ cells, do not require costimulatory signals from CD28 to acquire their effector fates in the thymus. Likewise, we have shown that, in an Lm infection model, γδ T cells differentiate into IL-17- and IFNγ-producing effectors, which contribute to bacterial clearance, independently of CD28 costimulation. This independence may be explained by enhanced glucose metabolism, a strong signal through the γδTCR, or both.

## Supporting Information

Figure S1 **Comparison of CD28 expression levels on γδ T cell subsets in the thymus and periphery of γδTCR Tg mice.** Analysis of CD28 expression on various gated subsets in the thymus (**A**) and pLNs (**B**) of CD28^+/+^ (i.e., IL-23R^gfp/+^) γδTCR Tg mice, Black histograms show representative staining of CD28 on total, IL-23R^+^ (γδ-17) and CD27^+^ (γδ-IFNγ) DN TCRγδ^+^ subsets. Staining of total thymocytes (**A**) and pLN cells (**B**) from CD28^−/−^ mice are shown as negative controls (shaded histograms). Numbers in the plots represent the mean fluorescent intensity (MFI) of CD28 expression. Data are representative of nine mice in four independent experiments.
(TIF)

Figure S2 **Effect of CD28 deficiency on γδ T cell cytokine production,** ***in vitro*** **and** ***in vivo*** **.** (**A**) Comparison of IL-17A and IFNγ production by anti-CD3 stimulated DN γδTCR^+^ cells from γδTCR Tg and TCRα^−/−^ mice in the presence or absence of anti-CD28 mAb. LN cells were in vitro stimulated with 5 µg/ml of hamster IgG, 5 µg/ml of anti-CD3 mAb or 5 µg/ml each of anti-CD3 and anti-CD28 mAbs. 16 h later, cells were harvested and cytokine production was assayed by intracellular (i.c.) flow cytometric analysis. Dot plots show representative i.c. staining for IFNγ versus IL-17A in gated DN γδTCR^+^ cells. Numbers in quadrants represent percentage of cells in that quadrant. Data shown are representative of at least 3 mice per genotype. (**B**) CD28^+/+^ and CD28^−/−^ mice were infected i.p. with 3×10^4^ CFU Lm and sacrificed on day 1 and 5 post infection to examine the γδ T cell response. Dot plots show representative i.c. staining for IFNγ and IL-17A on gated TCRγδ^+^ cells of CD28^+/+^ and CD28^−/−^ mice, *in vitro* re-stimulated with 5 µg/ml anti-TCRγδ mAb, at day 1 (n = 5 mice per genotype) and day 5 (n = 13 mice per genotype). Numbers in gate represent percentage of cells in that gate.
(TIF)

Figure S3 **Comparison of GLUT1 and GLUT3 expression levels on γδ lineage cells.** (**A**) GLUT1 (top panel) and GLUT3 (bottom panel) expression levels on DN γδ T cells from the pLNs of γδTCR Tg CD28^+/+^ (left panels) and γδTCR Tg CD28^−/−^ (right panels) mice. Staining of DP thymocytes from each genotype is also shown as a negative control. Data are representative of 3 to 6 mice per genotype. (**B**) GLUT1 (left panel) and GLUT3 (right panel) expression levels on CD4^+^ CD3^+^ thymocytes, CD8^+^ CD3^+^ thymocytes, and γδ thymocytes. Staining on DP thymocytes (shaded histogram) is also shown as a negative control. Data are representative of three independent experiments and 7 CD28^+/+^ mice.
(TIF)
