# Supplementary figures and images for: γδ T Cells Acquire Effector Fates in the Thymus and Differentiate into Cytokine-Producing Effectors in a Listeria Model of Infection Independently of CD28 Costimulation
Source: PLoS One. 2013 May 9;8(5):e63178. doi: 10.1371/journal.pone.0063178 (PMC3650071; doi:10.1371/journal.pone.0063178)

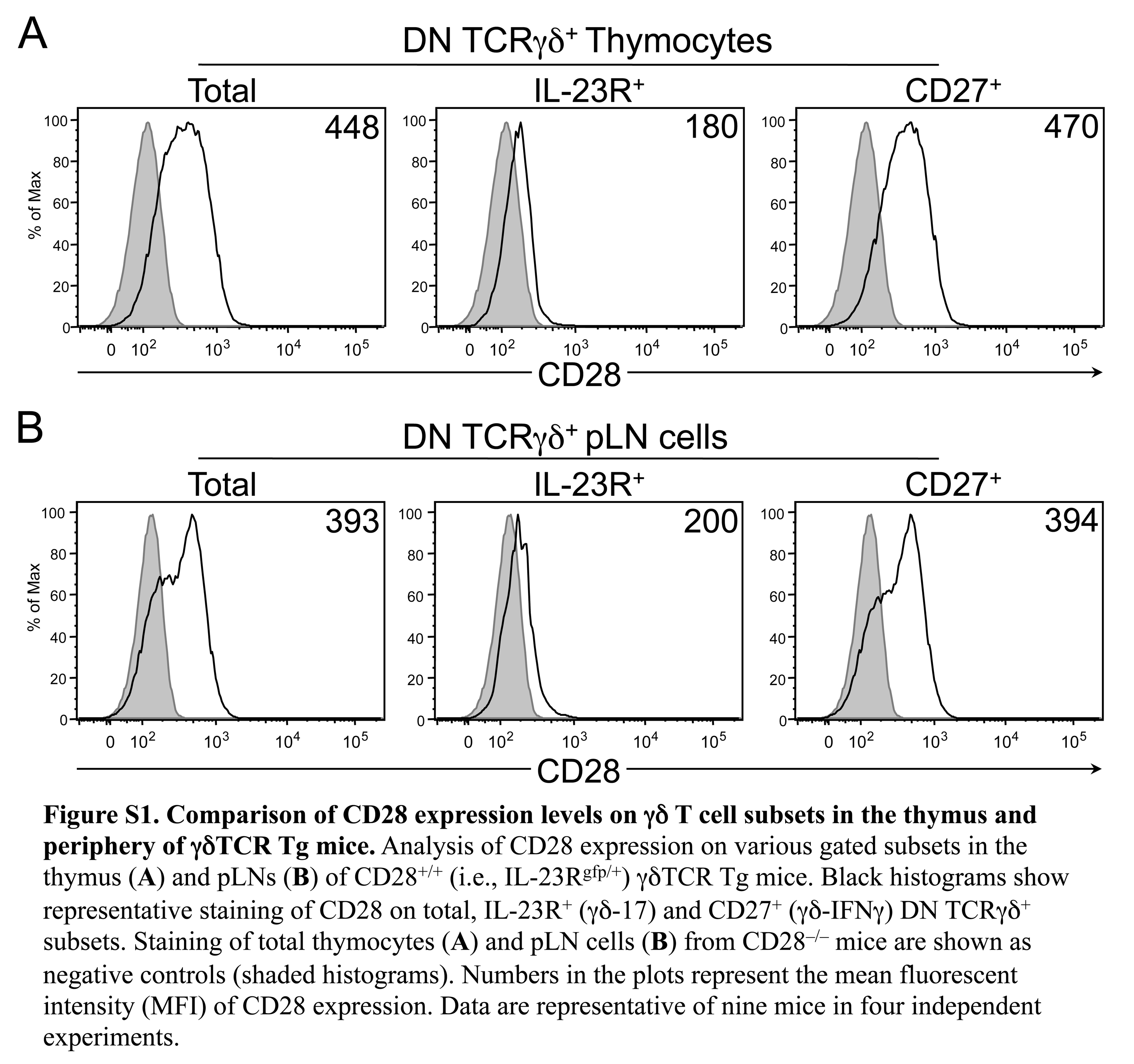

Supplement: Figure S1 — Comparison of CD28 expression levels on γδ T cell subsets in the thymus and periphery of γδTCR Tg mice. Analysis of CD28 expression on various gated subsets in the thymus (A) and pLNs (B) of CD28+/+ (i.e., IL-23Rgfp/+) γδTCR Tg mice, Black histograms show representative staining of CD28 on total, IL-23R+ (γδ-17) and CD27+ (γδ-IFNγ) DN TCRγδ+ subsets. Staining of total thymocytes (A) and pLN cells (B) from CD28−/− mice are shown as negative controls (shaded histograms). Numbers in the plots represent the mean fluorescent intensity (MFI) of CD28 expression. Data are representative of nine mice in four independent experiments. (TIF) [file pone.0063178.s001.tif]

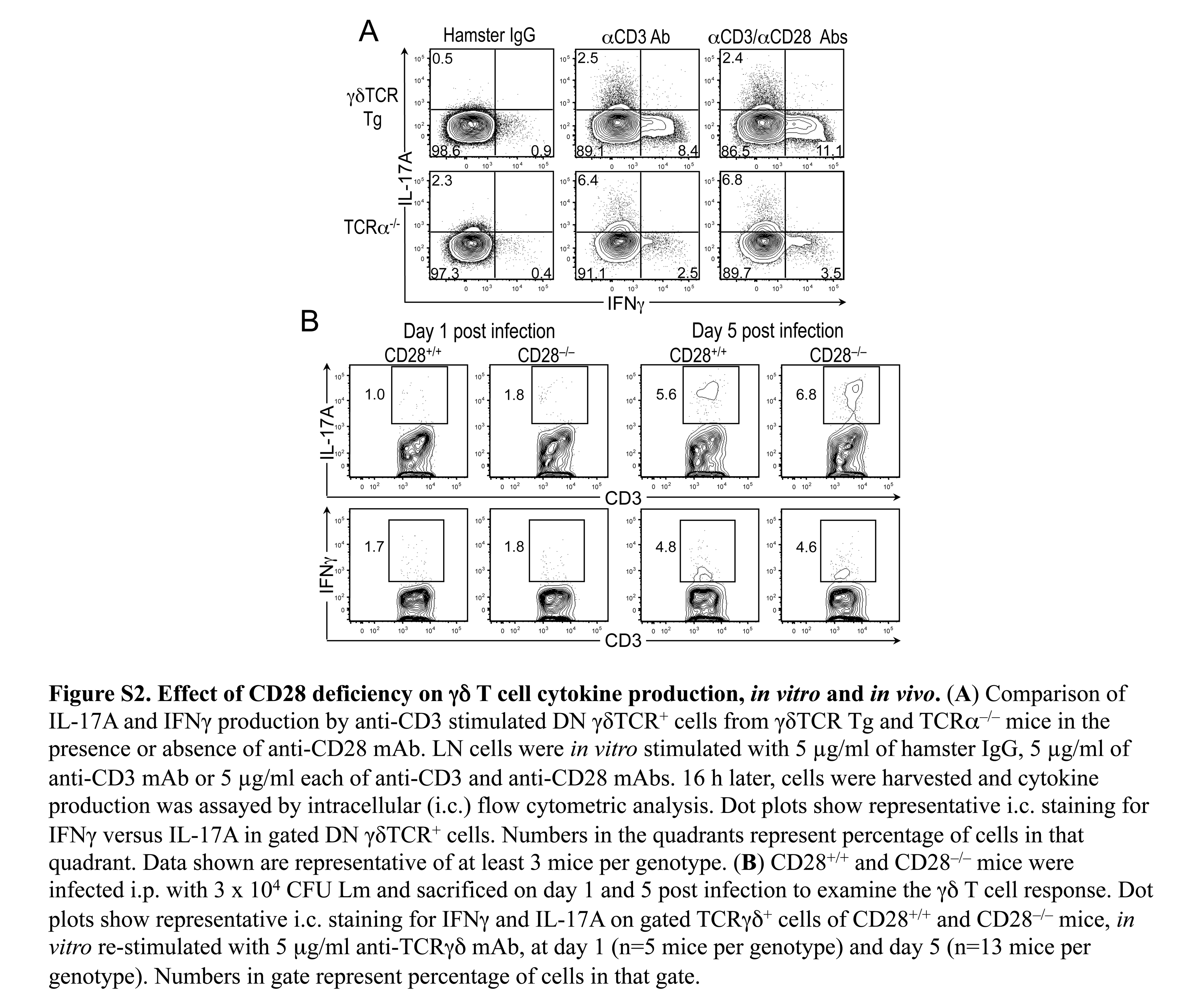

Supplement: Figure S2 — Effect of CD28 deficiency on γδ T cell cytokine production, in vitro and in vivo . (A) Comparison of IL-17A and IFNγ production by anti-CD3 stimulated DN γδTCR+ cells from γδTCR Tg and TCRα−/− mice in the presence or absence of anti-CD28 mAb. LN cells were in vitro stimulated with 5 µg/ml of hamster IgG, 5 µg/ml of anti-CD3 mAb or 5 µg/ml each of anti-CD3 and anti-CD28 mAbs. 16 h later, cells were harvested and cytokine production was assayed by intracellular (i.c.) flow cytometric analysis. Dot plots show representative i.c. staining for IFNγ versus IL-17A in gated DN γδTCR+ cells. Numbers in quadrants represent percentage of cells in that quadrant. Data shown are representative of at least 3 mice per genotype. (B) CD28+/+ and CD28−/− mice were infected i.p. with 3×10^4^ CFU Lm and sacrificed on day 1 and 5 post infection to examine the γδ T cell response. Dot plots show representative i.c. staining for IFNγ and IL-17A on gated TCRγδ+ cells of CD28+/+ and CD28−/− mice, in vitro re-stimulated with 5 µg/ml anti-TCRγδ mAb, at day 1 (n = 5 mice per genotype) and day 5 (n = 13 mice per genotype). Numbers in gate represent percentage of cells in that gate. (TIF) [file pone.0063178.s002.tif]

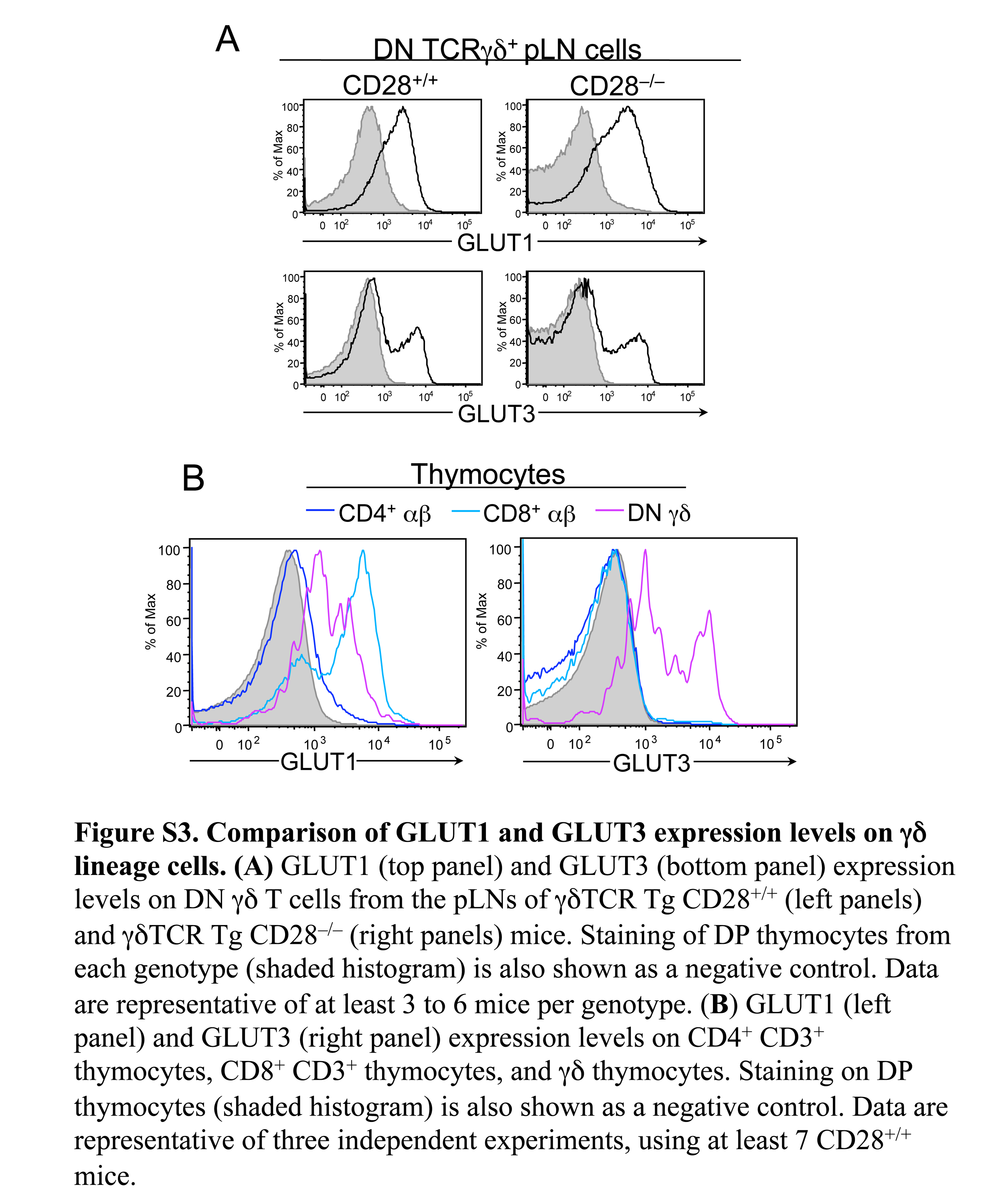

Supplement: Figure S3 — Comparison of GLUT1 and GLUT3 expression levels on γδ lineage cells. (A) GLUT1 (top panel) and GLUT3 (bottom panel) expression levels on DN γδ T cells from the pLNs of γδTCR Tg CD28+/+ (left panels) and γδTCR Tg CD28−/− (right panels) mice. Staining of DP thymocytes from each genotype is also shown as a negative control. Data are representative of 3 to 6 mice per genotype. (B) GLUT1 (left panel) and GLUT3 (right panel) expression levels on CD4+ CD3+ thymocytes, CD8+ CD3+ thymocytes, and γδ thymocytes. Staining on DP thymocytes (shaded histogram) is also shown as a negative control. Data are representative of three independent experiments and 7 CD28+/+ mice. (TIF) [file pone.0063178.s003.tif]
